# Implant removal rate after partial carpal arthrodesis in dogs: A retrospective analysis of 22 cases

**DOI:** 10.3389/fvets.2023.1160129

**Published:** 2023-04-04

**Authors:** Caroline J. Choi, Jason M. Balara, Sue A. Casale, Kirk L. Wendelburg

**Affiliations:** ^1^Department of Surgery, VCA Animal Specialty Group, Los Angeles, CA, United States; ^2^Department of Surgery, Angell Animal Medical Center, Boston, MA, United States

**Keywords:** carpus, hyperextension, arthrodesis, injury, implant

## Abstract

**Introduction:**

The purpose of this study is to determine the rate of implant removal after partial carpal arthrodesis and to investigate factors associated with implant removal.

**Methods:**

Case records of 22 dogs that underwent partial carpal arthrodesis at two private veterinary referral hospitals were reviewed. Details retrieved were body weight at time of surgery, sex, neuter status, breed, age, cause of carpal hyperextension injury, joint(s) involved in carpal hyperextension injury, laterality, type of implant, administration of post-operative antibiotics, post-operative outcome and indication for implant removal. Association between these factors and implant removal was evaluated.

**Results:**

Of 22 partial carpal arthrodesis, 12 (55%) had implant removal due to persistent lameness and 9/12 (75%) returned to full and acceptable function after implant removal. Indications for implant removal were implant interference (8), infection (4), and migration (1). When comparing type of implant, there was a significant difference when observing implant removal rates (*p* = 0.04). All 5 dogs with pins and wires (100%) required implant removal. Of 17 dogs with a plate, 7 (41.2%) required implant removal. Implant removal was performed on average 114 days post-operative.

**Discussion:**

Implant removal after partial carpal arthrodesis was frequent and was commonly indicated due to pin and wire fixation or plate implant interference. This study may impact how we prepare clients for potential post-operative complications and implant removal when recommending partial carpal arthrodesis.

## 1. Introduction

Partial carpal arthrodesis is considered a salvage surgical procedure for carpal hyperextension injuries that do not involve the antebrachiocarpal joint. Antebrachiocarpal joint injuries account for as low as 11–31% of cases, and a majority of hyperextension injuries involve either the middle carpal and/or carpometacarpal joint (1–3). The antebrachiocarpal joint is responsible for ~85% of the movement of the carpus and preserving its function may improve long-term outcomes (3).

Common techniques described for partial carpal arthrodesis include T-plates and cross pins for distal joint fusion. One study demonstrated satisfactory long-term results after application of a dorsal T-plate for middle carpal and carpometacarpal arthrodesis in a dog (4). Haburjak demonstrated multiple advantages of the use of cross pins in 23 carpi including technical ease, ability for greater latitude in pin placement, and ease of implant retrieval if necessary (5). Other reported surgical techniques for partial carpal arthrodesis include intramedullary pinning, dorsal dynamic compression plating, dorsal twin plating, and medial plating (1, 6–8).

Additional studies investigated long term outcomes of partial carpal arthrodesis. One study retrospectively evaluated canines that underwent partial carpal arthrodesis in 45 carpi and reported satisfying results from all clients based on questionnaires (6). Eleven percent of the dogs developed post-operative carpal hyperextension and 15.5% developed degenerative joint disease. These dogs still maintained adequate function of the limbs and did not require subsequent pancarpal arthrodesis (6). Additional studies compared long term outcomes of pancarpal and partial carpal arthrodesis in dogs. In one study, 74% of dogs treated with pancarpal arthrodesis regained full limb function and, although only 10 dogs were treated with partial carpal arthrodesis, 50% of these cases regained full limb function (1). In a clinical study, both pancarpal and partial carpal arthrodesis groups resulted in adequate limb function by demonstrating no difference in vertical, braking and propulsive gait parameters (9). However, propulsive forces and impulses were reduced in dogs that underwent pancarpal arthrodesis (9).

Common complications that have been reported in literature include continued persistent lameness by implant interference, implant failure, infection, and bandage morbidity (1, 5, 6). Many of these complications can be resolved with implant removal after bone healing is complete. Although different surgical techniques and post-operative long-term outcomes have been described for partial carpal arthrodesis, retrospective studies of post-operative complications requiring removal of implants are limited. The purpose of this study is to determine the rate of implant removal after partial carpal arthrodesis and to investigate factors associated with implant removal. Our first hypothesis was that there is no association between implant removal and the following factors: signalment, joint involvement, implant type, laterality, and administration of post-operative antibiotics. Our second hypothesis was that the most common indication for implant removal in patients with partial carpal arthrodesis is implant interference.

## 2. Materials and methods

### 2.1. Data collection

Case records of dogs that underwent partial carpal arthrodesis at VCA Animal Specialty Group, Los Angeles between July 1st, 2009 and July 1st, 2019 and at Angell Animal Medical Center between December 1st, 2011 and December 1st, 2021 were reviewed. Cases were included if at least 6 months of postoperative follow-up examination reports were available. For dogs that underwent bilateral partial carpal arthrodesis, initial procedure was included and subsequent surgery to the contralateral side was not included to remove statistical bias. Details retrieved were body weight at time of surgery, sex, neuter status, breed, age, cause of carpal hyperextension injury, joint(s) involved in carpal hyperextension injury, laterality, type of implant, administration of post-operative antibiotics, post-operative outcome and any implant removals. Joint(s) involved in carpal hyperextension injury were determined by radiographic review of plain and stressed radiographs by a board-certified radiologist. Additional details retrieved included indication for implant removal and post-operative outcome after implant removal. Postoperative outcomes were classified as persistent lameness or full/acceptable outcome based on definitions proposed by Cook and other (10).

### 2.2. Surgical treatment

Surgeries were performed in surgical suites by either a board-certified surgeon or surgical resident under direct supervision of a board-certified surgeon. Prior to surgery, all surgical sites were clipped and aseptically cleaned using a hanging limb technique. Cefazolin (22 mg/kg IV every 90 min throughout surgery) was administered.

Partial carpal arthrodesis was performed *via* a dorsal approach to the distal radius and carpus. Sufficient exposure to the radiocarpal bone, carpal bones, and metacarpal bones was obtained with care to avoid disturbance to the antebrachiocarpal joint. The intercarpal and carpometacarpal joints were opened, and the articular cartilage was removed to the level of the subchondral bone using a high speed burr. For each procedure, cancellous bone graft was retrieved from the ipsilateral humerus *via* a direct approach to the greater tubercle of the humerus using a curette. Autogenous cancellous bone graft was placed, and the joint was stabilized utilizing either a T-plate or Steinman pin and wires. Copious lavage with sterile saline was performed before closure.

For all cases, a caudal splint bandage was placed after the procedure for 4–8 weeks until there was evidence of clinical union. Clinical union was defined as a stable, pain-free carpus based on palpation with radiographic evidence of joint space bridging.

Decisions regarding administration of prophylactic postoperative antibiotics were determined by the primary surgeon. For cases that received antibiotics, postoperative antibiotics were prescribed for 10–14 days immediately following surgery. Antibiotics included cephalexin (22 mg/kg every 8 h), cefpodoxime (5–10 mg/kg every 24 h) and amoxicillin (13.75 mg/kg every 12 h). Sutures were removed at 10–14 days post-operative, and radiographs were performed 4–8 weeks postoperatively to assess bone healing.

### 2.3. Indications for implant removal

Implant removal was performed based on persistent lameness and radiographic evidence indicative of either implant interference, infection and/or implant migration. Implant interference was determined by radiographic review of fully extended and/or fully flexed views of the carpus. Dogs were categorized as requiring implant removal for infection only when confirmed by a positive microbial culture associated with the implant. Implant migration was diagnosed by evidence of implant displacement based on radiographic review.

### 2.4. Implant removal

Implant removal was performed *via* a direct approach to the carpus and metacarpus. Underlying tissues were debrided with blunt and sharp dissection until implants were exposed. The plate and its associated screws and/or pins and wires were removed. A culture was obtained at the surgeon's discretion and copious lavage was performed before routine closure.

### 2.5. Statistical analysis

Descriptive data analysis was used to characterize evaluated clinical features. These features included signalment, joint(s) involvement in hyperextension injury, implant type, laterality, administration of post-operative antibiotics, infection, implant migration, implant interference, implant removal rate and outcome of implant removal. Comparisons of the features and removal rate were by means of unpaired *t*-test or the Wilcoxon rank sum test (dependent on normality) for the continuous factors and Fisher's exact test or the Cochran Armitage test for categorical or ordinal factors, respectively. Normality was assessed by means of the Shapiro Wilk test. Frequencies with their 95% Wilson confidence limits were calculated and compared by means of the Wald Z score. *P* < 0.05 was considered significant. All calculations were by means of NCSS 2019 (Kaysville, UT).

## 3. Results

A total of 22 (9 left, 13 right) partial carpal arthrodesis surgeries were included for review. Twelve cases were excluded due to lack of follow up. Among 22 partial carpal arthrodesis, 12 (55%) had implant removals due to persistent lameness.

The most common breed was German Shepherd Dogs (*n* = 5) followed by Labrador crossbreeds (2). Fifteen other breeds were also represented. Gender distribution was 12 spayed females (54.5%), 7 neutered males (31.8%), 2 sexually intact females (9.1%) and 1 sexually intact male (4.5%). Mean age ± SD for patients requiring implant removal was 6.17 ± 2.37 years (range, 3–11 years). Mean age ± SD for patients without implant removal was 5.30 ± 2.00 years (range, 2–8 years). Mean weight ± SD for patients requiring implant removal was 29.7 ± 11.3 kg (range, 2.3–46.2 kg). Mean weight ± SD for patients without implant removal was 33.1 ± 16.2 kg (range, 1.8–54.4 kg). No significant difference was noted for implant removal when comparing sex, neuter status, weight, age, breed and laterality.

Fifty-five percent of the carpal hyperextension injuries was caused from either jumping or falling from a height, followed by unknown trauma (18%). Other causes included running into a ditch, chasing, traffic accident, and cage injury. Of 22 partial carpal arthrodesis, 7 (31.8%) involved injury to both the carpometacarpal joint and the middle carpal joint, 12 (54.6%) involved only the carpometacarpal joint, and 3 (13.6%) involved only the middle carpal joint. No significant difference was noted for implant removal based on the joint involved in hyperextension injury.

Postoperative antibiotics were administered to 10 dogs (45.5%) and the remaining 12 (54.6%) did not receive postoperative antibiotics. No significant difference was noted for implant removal based on administration of postoperative antibiotics.

All 5 dogs with pins and wires (100%) required implant removal. Of the 17 dogs where a plate was used, 7 (41.2%) required implant removal. Patients with pins and wires required implant removal significantly more frequently than patients with plates (*p* = 0.04).

Implant removals were performed at various times for each case. Details regarding the timeline of initial surgery and implant removal are summarized in Table 1. Overall, implant removal was performed on average 114 days post-operative.

**Table 1:** Case details of implant removals in 12 dogs.

**Dog**	**Breed**	**Age (y)**	**Sex**	**Indication**	**Implant removal (days post-operative)**	**Outcome (post-implant removal)**
1	Boxer mix	8	FS	IF	105	A
2	Viszla mix	6	FS	IN	79	A
3	German Shepherd	6	FS	IN	63	PL
4	Yorkshire Terrier	3	FS	M	91	A
5	Labradoodle	8	FS	IF	142	A
6	Pit Bull Terrier	4	FI	IF	72	A
7	Australian Shepherd	11	FS	IF	107	A
8	German Shepherd	8	MI	IF	22	A
9	Dutch Shepherd	7	FS	IF	103	A
10	German Shepherd	5	MN	IF and IN	133	FF
11	Labrador cross	5	MN	IF	235	PL
12	Siberian Husky	3	FS	IN	217	PL

Y, years; IF, interference; IN, infection; M, migration; A, acceptable; FF, full function; PL, persistent lameness.

Of the 12 patients requiring implant removal, the most common indication was implant interference (8/12, 67%), followed by infection (4/12, 33%), and implant migration (1/11, 8%). The one case with implant migration also had an infection. When comparing indications, interference was significantly associated with implant removal when compared to implant migration (*p* = 0.027).

Before implant removal, 14/22 (63.6%) had persistent lameness and 8/22 (36.4%) had full or acceptable function after partial carpal arthrodesis. After implant removal, 9/12 (75%) returned to full or acceptable function and 3/12 (25%) remained persistently lame.

## 4. Discussion

The results of this retrospective study revealed an implant removal rate of 55% in dogs that underwent partial carpal arthrodesis with pins and wires requiring removal significantly more frequently than plates and screws. In a previous study looking at partial carpal arthrodesis, only 9 of 45 cases required implant removal (20%) (6). The cases requiring implant removal all involve pin fixation although the number of patients with each implant type (T plate vs. pins) was not reported. In another study describing a cross pin technique, implant removal was performed in most patients at the time of radiographic union, unless owner declined, or retrieval required extensive exposure (5).

Implant removal was required in 8 of 43 pancarpal arthrodesis which demonstrates an implant removal rate of 18.6% (1). Studies on other common orthopedic procedures such as a tibial plateau leveling osteotomy (TPLO) demonstrated implant removal rates ranging from 2.7% to 7.4% (11–13). Based on these outcomes, our implant removal rate is substantially higher. Prevalence of implant removal may be due to variability in study population, implants used, and technical error. The number of cases lost to follow up may also influence our rate of implant removal. Type and duration of post-operative immobilization may also influence implant removal but was not evaluated in this study as all patients had splints placed post-operatively.

Although plates were more commonly used, dogs with pins and wires all eventually required implant removal, representing a significant association with implant type. This finding does not support our first hypothesis but may provide information on the benefit of using a plate over pins and wires. An *ex vivo* study analyzing the biomechanics of implants for partial carpal arthrodesis demonstrated T-plate's superiority to cross pinning in reducing intercarpal and carpometacarpal micromotion (14). Increased micromotion seen in cross pinning may promote implant migration or incomplete intercarpal fusion. These are common complications demonstrated by a case series of 21 partial carpal arthrodesis with cross pins (5). Alternatively, if a surgeon prefers to use pins and wires, client education may necessitate better preparation for the possibility of, or even requirement for, implant removal.

There appears to be no association between dogs that underwent implant removal and signalment or laterality. Most injuries in the present study were caused from jumping from a height, involving both the middle carpal joint and the carpometacarpal joint. No association between joint involvement and implant removal was noted. In our study, joint involvement was determined by radiographic review and physical examination. Because no patients in the present study required pancarpal arthrodesis because of poor outcome, the decision to perform partial carpal arthrodesis was supported.

Post-operative antibiotics were administered prophylactically, at the discretion of the primary surgeon. Ten of 22 cases received post-operative antibiotics, but there was no significant difference in implant removal rates based on administration of postoperative antibiotics. Further studies are required to justify the use of postoperative antibiotics.

**Figure 1:**
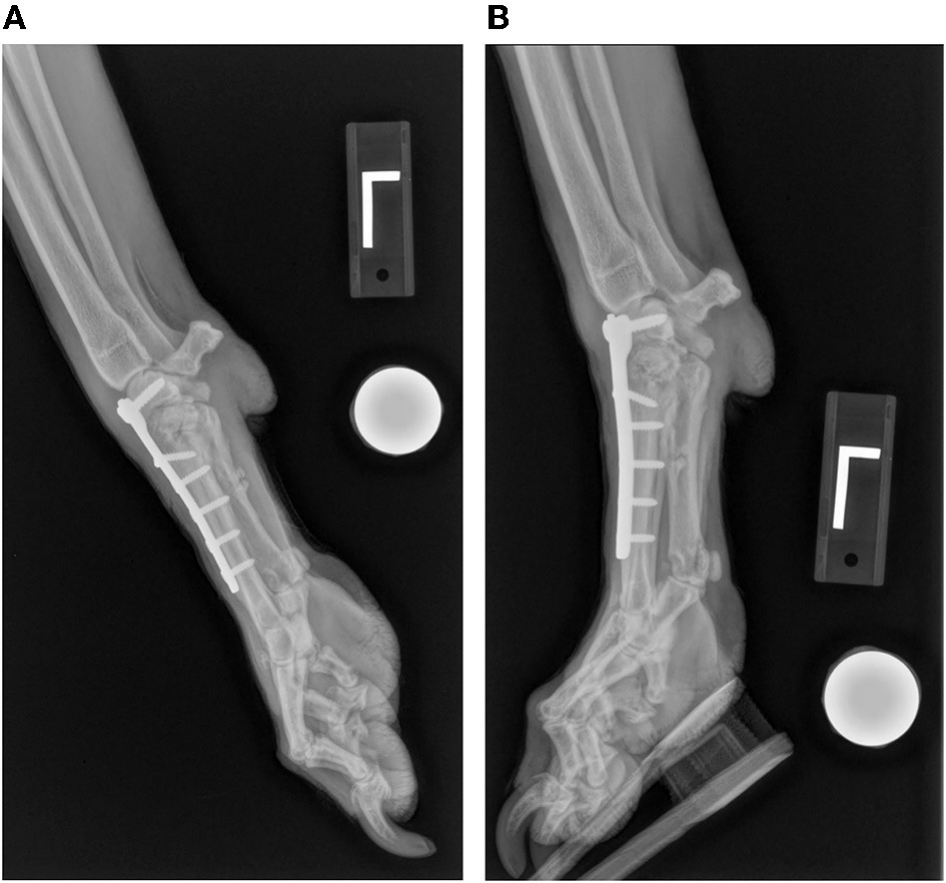
Post-operative partial carpal arthrodesis radiographic views of the left carpus. **(A)** Lateral view of the left carpus in neutral position showing appropriate plate and screw placement. **(B)** Lateral view of the left carpus in hyperextension showing implant interference between the proximal aspect of the plate and the radius.

**Figure 2:**
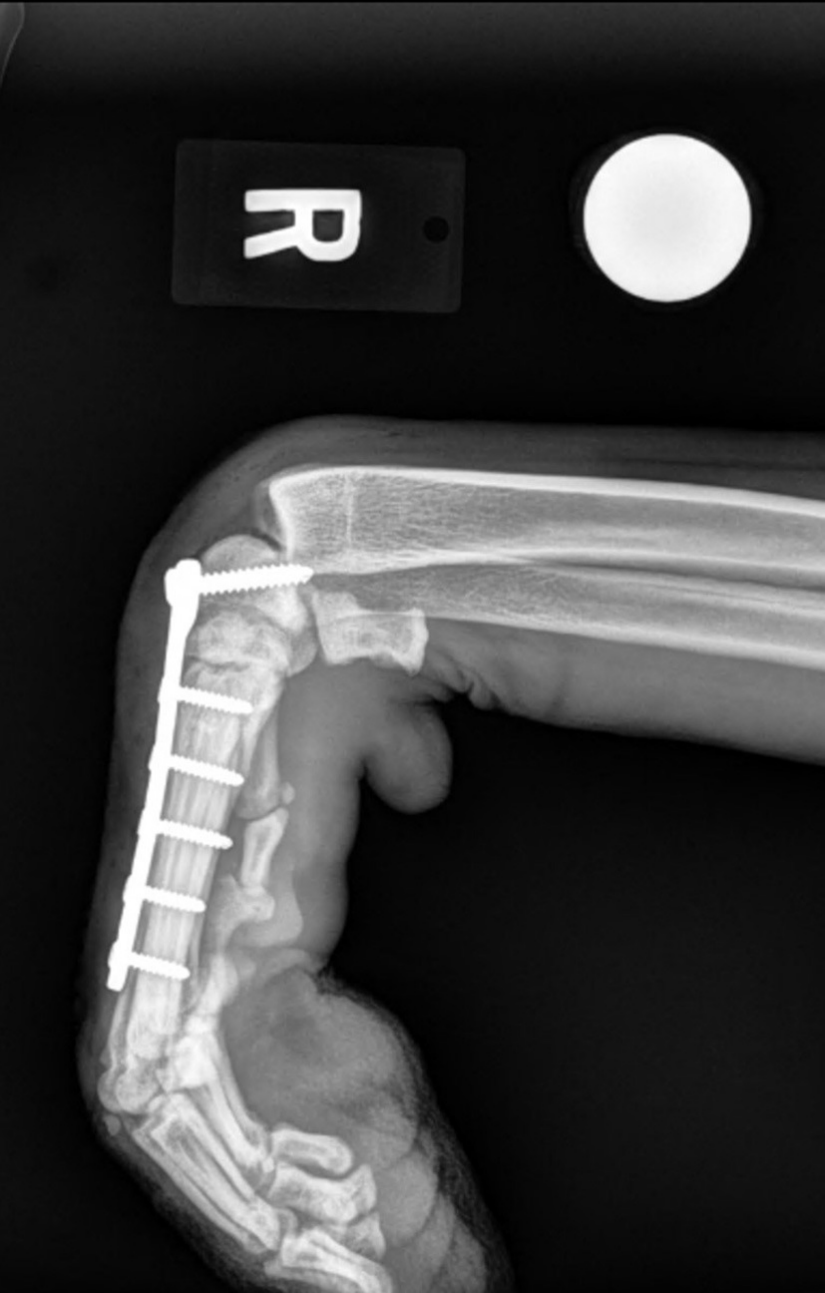
Post-operative partial carpal arthrodesis lateral radiographic view of the right carpus in hyperflexion showing implant interference between the most proximal screw and the radius.

**Figure 3:**
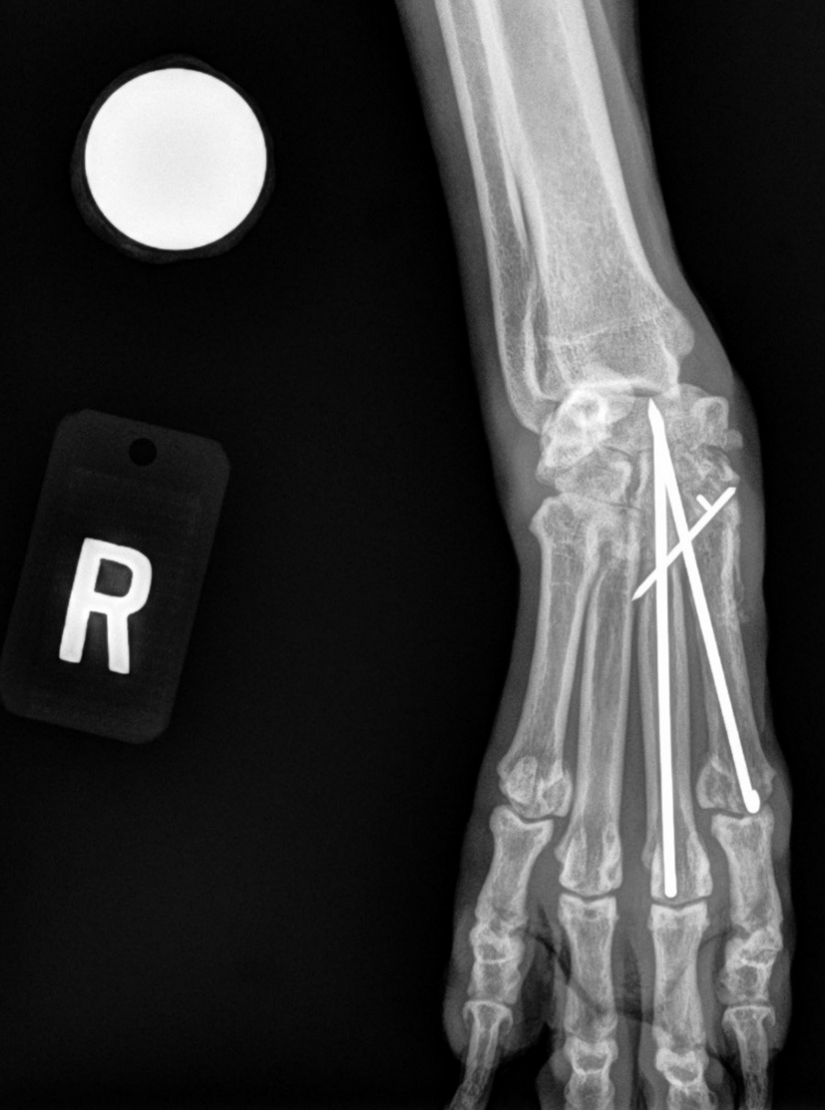
Post-operative partial carpal arthrodesis anterior to posterior view of the right carpus showing implant interference of the pin and antebrachiocarpal joint.

Of the 22 partial carpal arthrodesis, 63.6% had persistent lameness prior to patients undergoing implant removal. This value compares similarly with the outcome described by Denny et al.; however, in that study, implant removal was not discussed (1). In our study, implant removal was indicated due to persistent lameness caused by three specific reasons: implant interference, infection, and implant migration. These indications were also common postoperative complications reported in previous studies (1, 5, 6). Implant interference was the most common indication for implant removal, significantly more than implant migration. Thus, these findings allow us to accept our second hypothesis. Improvement in surgical technique to prevent implant interference may improve outcome and reduce the need for implant removal. Thorough surgical planning including premeasurement of plates and screws and implementing post-op stressed views to evaluate implant impingement should be considered. If available, use of advanced imaging intra-operatively with fluoroscopy may also allow improved visualization of implant placement. Post-op non-stressed lateral view of the left carpus shows appropriate placement of the plate and screws but does not show interference (Figure 1A). However, when the carpus is fully extended, the plate interferes with the radius (Figure 1B). Placing the plate as distal as possible below the joint surface and starting the screws at the most dorsal and distal surface of the radiocarpal bone may help avoid this interference. Figure 2 also shows another post-operative fully flexed view, and one proximal screw interferes with the radius, causing potential discomfort and lameness. Lastly, Figure 3 depicts a post-operative radiograph of a carpal partial arthrodesis with pins and wires, and reveals potential interference of the pin in the radiocarpal joint.

Post-operative arthrodesis angle of the carpus was not evaluated in the current study. Partial carpal arthrodesis may limit range of motion to a certain extent, but the arthrodesis angle should be in close proximity to the normal standing angle to avoid adverse effects. Proper implant placement may still result in interference if post-operative hyperextension exists. If the distal two carpal joints are appropriately fused and post-operative hyperextension remains, injury involving the antebrachiocarpal joint must be considered. Measurement of pre-operative arthrodesis angle with comparison to the contralateral carpal joint may allow better assessment of the joints involved and aid in surgical planning. Post-operative measurements may also help determine the likelihood of implant removal in the future. Owners should be warned that if hyperextension of the radiocarpal joint persists post-operatively, pancarpal arthrodesis may be indicated in the future.

Following implant removal, 75% of dogs returned to full or acceptable function. Although the implant removal rate for partial carpal arthrodesis is high, the overall outcome after implant removal was successful. For the remaining 25% of dogs who were persistently lame after implant removal, other causes should be considered including osteoarthritis, malunion, and other tendon and ligamentous injuries. These findings can influence how we educate clients when recommending a partial carpal arthrodesis by emphasizing the possibility for implant removal.

In the current study, healing progress of the arthrodesis was not evaluated. Utilizing a healing score system or a lameness scale could have provided additional information to better understand and improve outcomes. Given the nature of this surgical procedure and post-operative care, lameness scores were difficult to assess, especially with splint bandages present. In addition, reports in human literature reveal that radiographs alone do not provide accurate assessments of joint fusion (15). Ideally, computed tomography is the preferred diagnostic tool and significantly more reliable (15). A healing score system providing quantitative analysis of joint fusion utilizing radiographs and/or computed tomography specific to arthrodesis procedures may provide beneficial information for future studies.

This study has limitations, many of which are inherent to its retrospective nature. The sample size was small so it may not accurately represent the general population and may have prevented data from reaching statistical significance. More importantly, many cases were excluded from this study due to lack of follow up, which will affect our implant removal rate. For future studies, a larger population including additional institutions may enhance the quality of the data. Our inability to evaluate unrecorded variables that influenced a surgeon's and client's decision for implant removal may have affected our findings. For example, cultures were obtained at the surgeon's discretion for potentially infected cases. This may create a bias potentially undercounting the confirmed infections in this study. Lastly, our data was also restricted to specific variables. Other factors involved that may contribute to persistent lameness were not evaluated including intraoperative technique, duration of immobilization post-operative, bandage morbidity and degenerative joint disease.

In conclusion, the present study demonstrated that implant removal following partial carpal arthrodesis is common with significantly more cases with pins and wires requiring removal when compared to plates and screws. The results of this study may provide beneficial information on surgical planning and may impact how we prepare clients for post-operative complications and implant removal when a partial carpal arthrodesis is recommended.

## Data availability statement

The raw data supporting the conclusions of this article will be made available by the authors, without undue reservation.

## Author contributions

CC, JB, SC, and KW contributed to the conception and design of the study. CC organized the data and wrote the first draft of the manuscript. All authors contributed to manuscript revision, read, and approved the submitted version.
